# Some Account of the Late Dr. John Hennen, Deputy Inspector of Army Hospitals

**Published:** 1829-04-01

**Authors:** 


### Some Account of the late Dr. John Hennen, Deputy Inspector of Army Hospitals.

Dr. Hennen, the subject of the following biographical sketch, was born in 1779, at Castlebar, in the County of Mayo, Ireland, where his father followed the profession of a surgeon, and was a man of considerable professional, as well as general attainments. He commenced his chirurgical studies under his father, and completed his medical education at Edinburgh, at which place, very early in life, and before entering into the army, he married a Miss Malcolm. In 1800, he accompanied the memorable expedition destined for Egypt, under the command of General Sir Ralph Abercrombie, being then asisstant-surgeon in the 40th Regiment of Foot. This corps, however, having been detained at Malta, on its way to Egypt, Mr. Hennen continued to serve with it until its return to England. Some time after this, he was promoted to be surgeon of the 7th Garrison Battalion, then quartered in Ireland, and was afterwards removed to the 2d battalion of the 30th foot. With this corps he went to Gibraltar, and, subsequently, to Portugal; in which latter country, in consequence of recent and extraordinary political events in Spain, there was about to commence the first scene of a most sanguinary and protracted contest between the mighty powers of France and Britain, for the liberation of Continental Europe. Here an ample field was opened to him, and an opportunity afforded for the display and improvement of those qualities and energies, mental and bodily, for which he was afterwards conspicuous, and which failed not in the course of this arduous campaign to attract the notice of Dr. Macgrigor, then at the head of the medical staff in the Peninsula, and now the Director General, a situation which he fills with no less honour to himself than with advantage to the service. Mr. Hennen continued to serve throughout this long and varied war; never failing to profit by every occasion which presented itself to him, of adding to his practical knowledge, more particularly in that branch which afterwards formed the subject of his principal work, viz. military surgery: in the mean time, his appointment to the rank of a staff surgeon, and to the medical charge of a division of the army, gave him more extensive facilities for prosecuting this favourite object. In the short peace of 1814, having been placed upon the half-pay, he settled in practice at Dumfries in Scotland. In the following year, the return of Napoleon from Elba to France having occasioned the assembling of a British army in Belgium, he was called from his retirement, and ordered to Brussels by his friend and patron Sir James M'Grigor, who had recently been elevated to the office of Director General. It may be perhaps deemed superfluous to say that, in this short, though momentous campaign, Mr. Hennen's indefatigable zeal and exertions were always as manifest as his skill and experience were found beneficial, particularly in his attention to the wounded officers and men after the actions which closed with the far-famed and decisive battle of Waterloo. Soon after these operations, viz. in September following, he was promoted to the rank of a Deputy Inspector of Hospitals; and, upon the breaking up of the medical establishments in the Netherlands, he was placed upon the staff at Portsmouth, in February, 1816. The period of comparative repose which now ensued, enabled him to select, and to more seriously prepare, the materials for his publication, intituled, "*Principles of Military Surgery*," a work which he had for some time contemplated, but which he did not complete and publish until after his removal to Edinburgh, which took place in October, 1818. While stationed at that place, unrivalled as a medical school, he diligently attended the lectures and hospitals, at the same time that he cultivated the friendship of the professors of that university; to several of whom he publicly acknowledges the friendly assistance he received, while preparing the second edition of his work on Military Surgery, published in the year 1825. In August, 1820, he took his degree as Doctor of Medicine at Edinburgh, and, shortly after this, he gave a private course of lectures on military surgery, previously to his quitting the Scottish metropolis for service in the Mediterranean. Here the sphere of his duties was materially extended. He superintended the medical department in that quarter; first at Malta till April 1825; afterwards at Corfu; and, at the close of the same year, he was removed to Gibraltar, where he continued to serve till the 3d of November, 1828, on which day he unfortunately fell a victim to the fatal epidemic, then raging at that place, after a service of twenty-nine years, during the greater part of which time he was employed on active professional duties. It appears that, for two months before his death, his public labours, both night and day, had been prodigious, and greatly beyond his strength: his mind likewise had been exceedingly harrassed by the calamities daily happening around him; by the peculiar weight and responsibility of his situation, added to the illness of every member of his family who were present with him. This combination of afflicting causes, no doubt, aggravated the disease which attacked him in the evening of the 29th of October. It commenced with sickness and vomiting, followed by yellowness of the skin, and, on the third day, ischuria. This last symptom left him the succeeding day; and the fever abated, giving his friends some hopes of his recovery. In the evening of the 2d November, however, the disease had put on a more alarming aspect, and, at six o'clock in the morning of the 3d November, 1828, he expired at the age of forty-nine years, greatly, and, it would seem, not only universally but very sincerely regretted by all who knew him. To use the expressions of one of his friends, who writes from Gibraltar,—"There is hardly a person on the rock who did not look upon him as a friend; and the opinion all along of the governor and of every one capable of judging, has been uniform, that, fatal as the disease has been, but, for his (Dr. Hennen's) exertions, it would have been incalculably more so."

Besides his principal work on "Military Surgery," Dr. Hennen is the author of several papers on professional subjects, which appeared in various periodical publications. The first of these was one upon Hospital Gangrene, which appeared in one of the early numbers of the "Medical Repository." His writings abundantly testify, that he held no ordinary rank as a surgeon; and that his mind was ardent, industrious, and zealous, in the improvement of his profession.

P.S. We are informed that a subscription is going on to erect a monument in Gibraltar, where a considerable sum has been subscribed by all classes, from the governor downwards. Besides the subscription paper in the Army Medical Board Office, subscriptions are going on in Dublin and in Edinburgh. It is notified by the Committee at Gibraltar, "that they entertain strong hopes, that, after erecting the monument, there will remain a sum worthy of the acceptance of Dr. Hennen's highly gifted daughter."

